# Pharmacokinetics, Tissue Distribution and Therapeutic Effect of Cationic Thermosensitive Liposomal Doxorubicin Upon Mild Hyperthermia

**DOI:** 10.1007/s11095-015-1815-y

**Published:** 2015-10-30

**Authors:** Bilyana M. Dicheva, Ann L. B. Seynhaeve, Thomas Soulie, Alexander M. M. Eggermont, Timo L. M. ten Hagen, Gerben A. Koning

**Affiliations:** Laboratory Experimental Surgical Oncology, Section Surgical Oncology, Department of Surgery, Erasmus Medical Center, POBox 1738, 3000 DR Rotterdam, The Netherlands; Institut Gustave Roussy, Villejuif, France

**Keywords:** cationic thermosensitive liposomes, doxorubicin, hyperthermia, therapeutic efficacy

## Abstract

**Purpose:**

To evaluate pharmacokinetic profile, biodistribution and therapeutic effect of cationic thermosensitive liposomes (CTSL) encapsulating doxorubicin (Dox) upon mild hyperthermia (HT).

**Methods:**

Non-targeted thermosensitive liposomes (TSL) and CTSL were developed, loaded with Dox and characterized. Blood kinetics and biodistribution of Dox-TSL and Dox-CTSL were followed in B16BL6 tumor bearing mice upon normothermia (NT) or initial hyperthermia conditions. Efficacy study in B16BL6 tumor bearing mice was followed with Dox-TSL or Dox-CTSL upon NT or HT. Efficacy study in LLC tumor bearing mice was performed upon two HT conditions. Intravital microscopy was performed on B16BL6 tumors implanted in dorsal-skin fold window-bearing mice.

**Results:**

Targeting did not cause faster blood clearance of CTSL compared to TSL. Highest uptake of liposomes was observed in spleen, kidneys and liver. Applying HT prior to CTSL administration increased drug delivery to the tumor and CTSL delivered ~1.7 fold higher Dox concentration compared to TSL. Efficacy in B16BL6 murine melanoma showed that HT had a significant effect on CTSL in tumor suppression and prolonged survival. Efficacy in LLC Lewis lung carcinoma tumor model demonstrates that two HT treatments hold promises for a successful treatment option.

**Conclusion:**

CTSL have potency to increase drug efficacy in tumors due to their targeted and drug release functions.

## Introduction

Liposomes as one of the best studied nanocarriers for treatment of cancer improve pharmacokinetics and biodistribution of the encapsulated chemotherapeutic drugs after systemic administration (1). Despite their prolonged blood circulation, in the clinic, pegylated limosomal doxorubicin has only limited therapeutic efficacy (2,3) due to its low tumor retention and low drug bioavailability (4–6). An idea to improve this includes targeting of liposomes with specific ligands for increased tumor retention together with an external trigger, *i.e.,* heat, which can increase drug delivery locally in the tumor area while preventing the healthy tissues from side effects. The aim of this study was to use cationic thermosensitive liposomes (CTSL) (7) loaded with doxorubicin (Dox), which combine both targeted and triggered characteristics of liposomes in one carrier in order to deal with the drawbacks of the liposomal chemotherapy and follow their behaviour *in vivo*. The designed nanoparticles made use of shielded cationic lipids for specific recognition of tumor vasculature and tumor cells in combination with thermosensitive lipid bilayers for heat-triggered drug release.

In the clinic, mild hyperthermia (HT) is known to increase the effect of chemo- and radiotherapy leading to enhanced therapeutic efficacy in cancer patients (8,9). Mild hyperthermia can inhibit DNA repair, augment tissue oxygenation and sensitize cancer cells to cytotoxic drugs (10,11). Additionally, HT is able to increase blood flow and interstitial fluid flow helping an enhanced passive perfusion of small molecules. More importantly, HT can increase nanoparticle extravasation by increasing the gaps between the vascular endothelial cells (12–14). In addition, HT can trigger drug release locally in the tumor (13,15–18). Previous studies have shown increased therapeutic effect from thermosensitive liposomes (TSL) triggered with mild HT. The effect was mostly due to extravasation and increased drug release locally in the tumor (18–22).

Another approach for improved drug bioavailability comes from active targeting of liposomes to the tumor. Decorating liposomes with ligands specific for tumor vasculature or tumor cells may result in their higher retention in tumors and subsequently increased drug delivery.

Cationic liposomes are known to specifically bind angiogenic endothelial and tumor cells due to the increased expression of negatively charged molecules on these cell membranes (23). The slower and irregular blood flow in tumors also promotes binding between passing cationic liposomes and tumor vasculature (24). The specific binding of CTSL to either endothelial or tumor cells may lead to receptor-mediated endocytosis of the carrier, therefore bringing the drug closer to the nucleus. CTSL are also composed of thermosensitive lipids with a large capacity to encapsulate drugs and release them upon heat. When HT is applied, CTSL lipid membrane undergoes gel-to-liquid crystalline phase transition and becomes more permeable towards water and solutes (25). In this way, the encapsulated hydrophilic drugs can be released intracellularly.

The therapeutic effect of HT together with TSL was investigated in many preclinical studies (19,22,26–35). A major drawback of these studies is the lack of uniform experimental setup, conditions and read-out.

Several TSL formulations have been studied (36). Al-Jamal *et al.* reported detailed on pharmacokinetics and biodistribution of different TSL in presence or absence of HT showing that TSL stability and make-up affect drug delivery and intratumoral availability (37). An optimum HT protocol requires knowledge on pharmacokinetics, biodistribution and tumor accumulation of the liposomal nanocarriers as HT may become a treatment option for many types of cancer. However, detailed understanding of the pharmacological behaviour of targeted thermosensitive liposomes is not available yet.

In this study, the pharmacokinetics, biodistribution and therapeutic efficacy of doxorubicin encapsulated in cationic thermosensitive liposomes (CTSL) were investigated. For the efficacy studies, we tested the tumor growth of two different types of tumors - B16BL6 murine melanoma and LLC Lewis lung carcinoma over time. The tumor growth of two groups—TSL and CTSL was studied with or without HT in B16BL6 or with two HT treatments in LLC . PBS was used as a control.

## Materials & Methods

### Chemicals

The phospholipids 1,2-dipalmitoyl-sn-glycero-3-phosphocholine (DPPC), 1,2-distearoyl-sn-glycero-3-phosphocholine (DSPC), 1,2-distearoyl-sn-glycero-3-phosphoethanolamine-N-PEG2000 (DSPE-PEG2000) were purchased from Lipoid (Ludwigshafen, Germany). The cationic lipid 1,2-dipalmitoyl-3-trimethylammonium-propane (DPTAP Chloride salt) was from Avanti Polar Lipid Inc. Doxorubicin-HCl was purchased from Pharmachemie (Haarlem, The Netherlands). Sodium 3′-[(1-phenylaminocarbonyl)-3,4-tetrazolium]-bis(4-methoxy-6-nitro)benzene sulfonic acid hydrate (XTT) was purchased from Sigma-Aldrich (Zwijndrecht, The Netherlands). Dioctadecyl tetramethylindotricarbocyanine perchlorate (DiD-C_18_(3)) was purchased from Invitrogen.

### Preparation of TSL

CTSL were composed of DPPC:DSPC:DPTAP:DSPE-PEG2000 in a molar ratio 62.5:25:7.5:5. TSL consisted of DPPC:DSPC:DSPE-PEG2000 in a molar ratio 70:25:5 All the liposomes were prepared by lipid film hydration and extrusion method. The lipids were dissolved in chloroform and methanol (9:1 vol/vol). Liposomes used for intravital microscopy contained 0.3% of DiD. The solvent was subsequently evaporated under vacuum in rotary evaporator until homogeneous lipid film was formed. The lipid film was hydrated in 250 mM (NH_4_)_2_SO_4_ solution at 60°C for 30 min. The newly formed multilammelar vesicles were extruded subsequently five times through 100 nm, five times through 80 nm and five times through 50 nm polycarbonate filter (thermo barrel extruder at 60°C) and resulted in small sized TSL. Extraliposomal (NH_4_)_2_SO_4_ was removed from liposomal (NH_4_)_2_SO_4_ by gel permeation chromatography using a PD-10 Sephadex column (GE Healthcare, Buckinghamshire, UK), eluted with HEPES buffer, pH 7.4 (10 mM HEPES, 135 mM NaCl). Size, polydispersity index (PDI) and zeta potential (ζ) were measured by dynamic light scattering using Zetasizer Nano ZS (Malvern Instruments, Worcestershire, UK). For size and PDI measurements, TSL were diluted in HEPES, pH 7.4, while the zeta potential was obtained in HEPES, pH 7 without NaCl. Lipid concentration was determined by phosphate assay (38). After the phosphate concentration was determined, doxorubicin was loaded into the liposomes (5 mM lipid) in 0.05:1 drug:lipid ratio (mol:mol) at 38°C for 1 h. The liposomes were concentrated by ultracentrifugation for 2 h, 4°C. The pellet was resuspended in HEPES buffer, pH 7.4 and left overnight on slow rotation at 4°C. Then the liposomes were passed through PD 10 column eluted with HEPES buffer, pH 7.4 to remove residual nonentrapped doxorubicin. Doxorubicin concentration was measured by spectrophotometer at Ex 480 nm.

### Cell Lines and Culture

Tumor cell lines B16 (murine melanoma) and LLC (Lewis lung carcinoma) were cultured in a Dulbecco’s Modified Eagles’ medium (Lonza, Belgium) containing 10% FCS. Cells were subcultured once a week using Trypsin (Sigma, Aldrich) and maintained at 37°C, 5% CO_2_ in a humidified incubator. All experiments were performed at a confluence of 80–90%.

### Animal Models

The eNOStag-GFP mice line in which the endothelial cells are visible due to constitutive expression of a GFP eNOS-tag fusion protein was used for intravital imaging. Mice weighing about 25 g were used and fed a standard laboratory diet *ad libitum* (Hope Farms Woerden, The Netherlands). All animal experiments were done in accordance with the Dutch law and protocols were approved by the committee on animal experimentation of the Erasmus MC, Rotterdam, the Netherlands.

Preparation of the dorsal skin-fold chamber with B16BL6 tumor is an adaptation from previously described procedures (13,18,19). The mice were housed in an incubation room with an ambient temperature of 30°C and a humidity of 70%. Experiments started 8 to 12 days after tumor implantation, at which a functional vasculature is established in the tumor. For *in vivo* efficacy study, ~ a 3 mm^3^ tumor piece of either B16BL6 or LLC tumors was implanted in the hind limb of C57BL6 mice. Mice were used for experiments when tumors reached ~5 mm in diameter.

### Intravital Microscopy for Dox and Liposome Retention in B16BL6 Tumors

DiD-labelled TSL or CTSL containing Dox were injected i.v. (5 mg/kg Dox) and let to circulate in the blood stream for 5 h in order to allow for liposome targeting to tumor vasculature. After the targeting phase, HT at 42°C for 1 h was applied to trigger drug release from the liposomes. Mice were observed by confocal microscopy (Zeiss LSM 510 META) up to 120 h after injection of Dox-TSL or Dox-CTSL in order to visualize Dox and liposome clearance from the tumor. Images of 1024 × 1024 pixels were analyzed using Zeiss LSM image software (Zeiss, Germany), 10× objective lens. Doxorubicin fluorescence was detected by a 543 nm Helium –Neon laser and DiD fluorescence by 613 nm nm Helium –Neon laser.

### Pharmacokinetic and Biodistribution of Dox-TSL and Dox-CTSL

Pharmacokinetics and biodistribution of Dox-TSL and Dox-CTSL were followed in B16BL6 tumor bearing mice upon NT or initial HT conditions. At NT condition, mice were injected with 3 mg/kg Dox and blood sampling was performed at 0.1;1;2;4;6 and 24 h and organs were collected 24 h after liposome injection. At HT condition, tumors were first preheated for 1 h at 41°C and then cooled down for 15 min, in order to facilitate liposome extravasation. Then, liposomes were injected at 3 mg/kg Dox and blood samples were collected up to 24 h (0.1;1;2;6;24 h), after which the organs were removed. Blood samples (~50 μL/sample), pieces of organs (~100 mg/organ) and complete tumors obtained during the blood kinetics and biodistribution experiments were analyzed for their doxorubicin concentrations. To all samples, an aqueous solution of daunorubicin (0.5 μg/mL in 1.5 mL H_2_O) was added as an internal standard for doxorubicin quantification, followed by homogenization for 5–20 min at 30 Hz in a Qiagen Tissuelyser. In order to extract doxorubicin, 125 μL of the homogenized blood and tissue solutions was incubated with 50 μL AgNO_3_ in water (33% *w*/*v*) for 10 min at room temperature. Subsequently, the doxorubicin was extracted by vigorous mixing with 1.25 mL chloroform/isopropanol (2:1 *v*/*v*). After centrifugation (10 min at 3600 rpm) the organic phase was transferred to a clean tube and evaporated to dryness at 40°C under N_2_ flow. The residue was dissolved in H_2_O (200 μL) of which 50 μL was injected onto the HPLC column. HPLC analysis was performed on an Agilent Technologies system (1100 series) equipped with an autosampler and fluorescence detector (λ_ex_ = 485 nm and λ_em_ = 590 nm). An Eclipse XDB-C18 column (5 μm, 4.6 × 150 mm^2^ Agilent) was used. The doxorubicin and daunorubicin were eluted in 6 and 12 min respectively, using an isocratic flow of 1 mL/min with 30% (*v*/*v*) acetonitrile in H_2_O containing 0.1% TFA (*v*/*v*). The Dox concentration in blood and organs was calculated as % injected dose/g tissue (%ID/g). Six mice were used per each group.

### Therapeutic Efficacy of Dox-TSL and Dox-CTSL in B16BL6 and LLC Tumors

C57Bl6 mice were implanted s.c. with B16BL6 murine melanoma or murine LLC Lewis lung carcinoma in their hind limbs. When tumor size reached 5 mm in diameter, mice were anesthetized and the tumor bearing hind legs except the tumor were covered with vaseline to protect them from direct heat. The tumor was in direct contact with the water bath. The hind legs were fixed on a rack to ensure a steady position in a water bath during the HT treatment. Thermocouples were attached to the tumor surface at multiple spots to monitor tumor temperature over time. The water bath temperature was set to 43°C to reach tumor temperature at 42°C. In B16BL6 bearing mice, mice were injected with PBS, Dox-TSL and Dox-CTSL (3 mg/kg Dox) and 5 h later HT at 42°C for 1 h was applied to trigger drug release. Mice with PBS, Dox-TSL and Dox-CTSL under NT were used as control groups. In LLC bearing mice, there was an initial HT treatment for 1 h at 41°C followed by a cool down for 15 min. Then, liposomes were injected and allowed to circulate for 5 h. A second HT for 1 h at 42°C was then applied to trigger drug release. After the treatment, the mice were returned back to the cages. The tumor size and the body weight were measured on the day of the experiment and every other day after the treatment. Mice were sacrificed if the tumor weight exceeded 10% of the body mass, the mice lost 10% body weight, when the tumor reached a tumor size of 1350 mm3 or at the end of the experiment.

### Histology

Mice implanted s.c. with murine B16BL6 melanoma were injected with 3 mg/kg Dox-TSL or Dox-CTSL and liposomes were allowed to circulate for 5 h. Then, HT for 1 h at 42°C was applied to trigger drug release. Organs and tumors were taken out 24 h after liposome injection. PBS without HT was used as a control.

### Statistics

*In vivo* biodistribution study was analyzed by Mann–Whitney test and results with *p*-value ≤ 0.05 were considered statistically significant.

## Results

### Pharmacokinetics and Biodistribution of Dox-TSL and Dox-CTSL

**Fig. 1:**
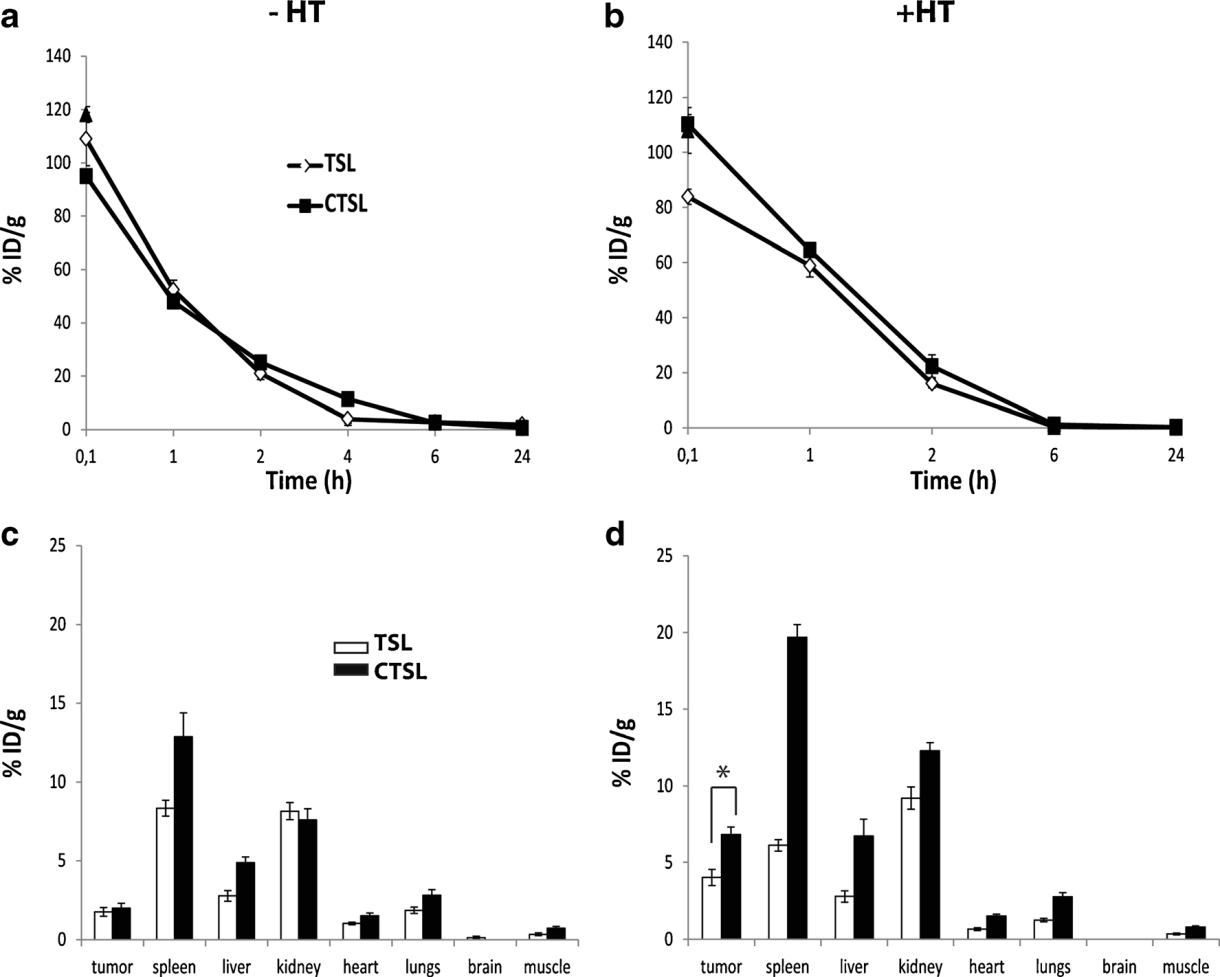
Pharmacokinetics (**a** and **b**) and biodistribution (**c** and **d**) of Dox-TSL and Dox-CTSL in B16BL6 tumor bearing mice upon NT or initial HT conditions. At NT condition (**a** and **c**), mice were injected with 3 mg/kg Dox and blood sampling was performed at the indicated time points and organs collected 24 h after liposomes injection. At HT condition (**b** and **d**), tumors in mice were preheated for 1 h at 41°C and cooled down for 15 min, in order to allow for liposome extravasation. Then, liposomes were injected at 3 mg/kg Dox and blood samples were collected up to 24 h, after which the organs were removed. The Dox concentration in the blood and organs was analyzed by HPLC. Six animals were used per group. *Mann–Whitney test, *p*-value ≤0.05.

The characteristics of the liposomes used here have been reported previously (7). In order to understand Dox clearance from circulation and its distribution in healthy organs and tumors, pharmacokinetic and biodistribution profiles of Dox in TSL or CTSL were followed (Fig. 1a and b) under NT or HT conditions. At both NT and HT conditions, the trend of Dox-TSL and Dox-CTSL clearance from circulation was similar. At NT condition (Fig. 1a), Dox from TSL and CTSL seemed to clear from circulation fast in the first 1 h (52 and 47% remaining Dox respectively). After 2 h of liposome circulation, there were ~20% remaining Dox from both formulations. At later time points (4, 6, 24 h) there was barely any Dox present in circulation from TSL whereas there were 11% Dox left from CTSL after 4 h of circulation. Considering the biodistribution of Dox (Fig. 1c and d), at both NT and HT conditions, there was a significant uptake of Dox from the two formulations in spleen as it was significantly higher for Dox from CTSL than TSL under HT conditions (19.7 v/s 6% ID/g). Similar high Dox accumulation in the kidneys was observed from the two formulations, which was slightly increased upon HT conditions for TSL but significantly increased for CTSL (7.6 to 12.4%ID/g). Dox accumulated in the liver was slightly higher for CTSL than for TSL under NT (4.7%ID/g v/s 2.7%ID/g respectively). However, under HT conditions there was an increase in delivered Dox from CTSL to the liver than TSL (6.7% ID/g v/s 3.7%ID/g respectively). The higher Dox uptake in spleen and liver from CTSL is due to most probably opsonization of CTSL by proteins in these organs. There was a minimal uptake of Dox from TSL and CTSL in the heart, lungs and muscle upon NT and HT. No Dox was detected in the brain from neither of the formulations. At NT, the tumor uptake of Dox was similar for the two formulations. However, the application of initial HT for 1 h at 41°C was able to cause ~2.3 fold increased Dox amount to the tumor from TSL (1.7 v/s 4% ID/g) and 3.4 fold increased Dox to the tumor from CTSL (2 v/s 6.8%ID/g). The initial HT treatment was able to significantly increase (1.7 fold) Dox delivery to tumor from CTSL compared to TSL.

### Tumor Growth Control and Survival of Mice with B16BL6 Tumors

**Fig. 2:**
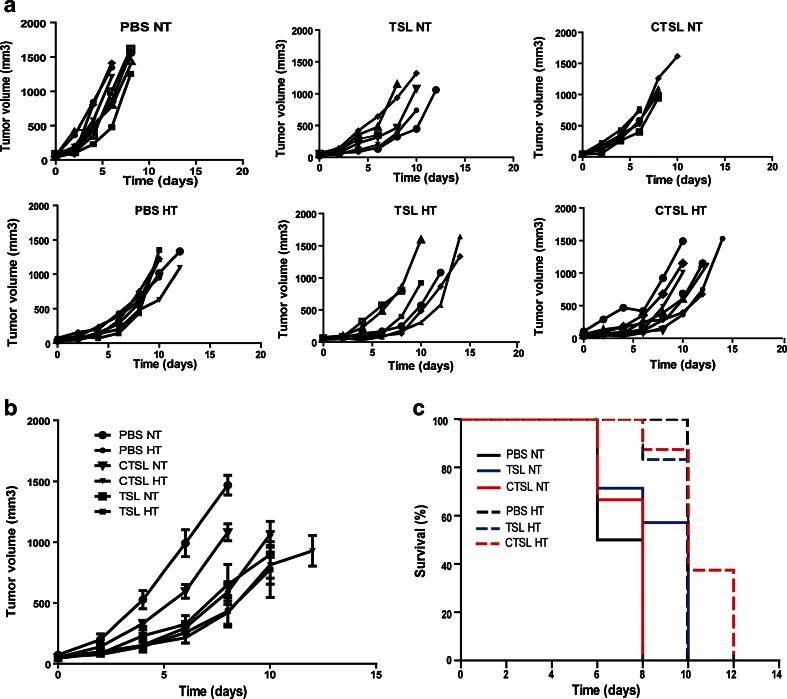
Mice implanted with B16BL6 tumors were injected with 3 mg/kg Dox-TSL or Dox-CTSL. In the HT group, liposomes were allowed to circulate for 5 h, after which HT at 42°C for 1 h was applied to trigger drug release. (**a**) Individual tumor growth curves from all mice in all treatment groups. (**b**) Efficacy of all treatments. (**c**) Survival of mice upon different treatments.

The efficacy of either Dox-TSL or Dox-CTSL was followed in B16BL6 tumor bearing mice upon either NT or HT conditions (Fig. 2a and b). HT itself showed a tremendous effect on tumor growth (A and B). HT effect on the tumor growth was comparable to TSL without HT. Interestingly, HT added to CTSL decreased significantly tumor growth compared to their effect on the tumor growth without HT. HT applied to TSL did not add to inhibiting tumor progression. However, the combination of liposomes and HT showed the highest therapeutic effect. In the CTSL plus HT group, four out of eight mice survived 12 days post-treatment whereas in the TSL plus HT group, five out of six mice survived 10 days post-treatment (C). In comparison, the group with CTSL without HT survived only 8 days post-treatment (four out of eight mice). Therefore, HT applied to mice treated with CTSL increased their survival by 4 days. HT added to TSL did not increase mice survival. HT added to PBS increased survival only with 1 day.

### Tumor Growth Control and Survival of Mice with LLC Tumors

**Fig. 3:**
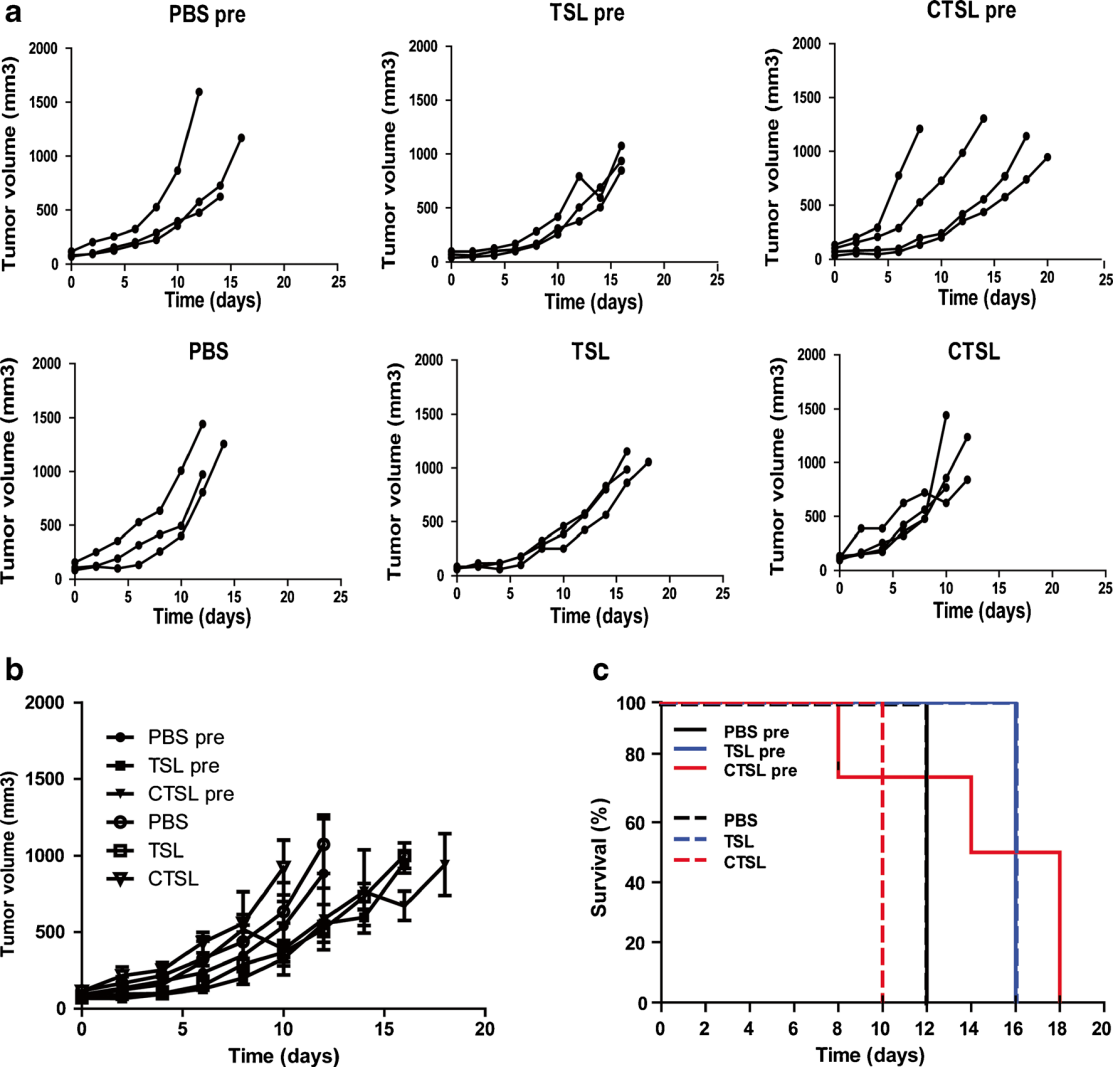
Mice implanted with LLC tumors were either preheated for 1 h at 41°C followed by 15 min of cooling down to body temperature or were not preheated. Then, they were injected with 3 mg/kg Dox-TSL or Dox-CTSL, after which the liposomes were allowed to circulate for 5 h at NT. After that, HT at 42°C for 1 h was applied to trigger drug release. (**a**) Individual tumor growth curves from all mice in all treatment groups. (**b**) Efficacy of all treatments. (**c**) Survival of mice upon different treatments.

The efficacy of Dox-TSL and Dox-CTSL was followed in mice implanted with LLC tumor model based on two different treatment schedules:—preheating of the tumor for 1 h at 41°C followed by cooling it down for 15 min, injection of liposomes, allowed to circulate for 5 h and subsequently application of HT for 1 h at 42°C or:—the same treatment skipping the preheating phase (Fig. 3). The preheating phase was used to induce extravasation of liposomes. Treatment with initial HT additionally to one HT treatment decreased significantly tumor growth and prolonged survival only in the case of Dox-CTSL (Fig. 3a and b). Survival was increased with 8 days (from 10 to 18 days). The effect on tumor growth of Dox-TSL and Dox-CTSL with preheat were similar. Dox-CTSL with preheat increased the survival of mice with 2 days (from 16 to 18 days) compared to Dox- TSL with preheat. Preheating phase did not add to one HT treatment in inhibiting tumor growth or increasing survival when Dox-TSL or PBS alone were used.

### Control on Treatment Toxicity in Mice with B16BL6 Tumors and LLC Tumors

**Fig. 4:**
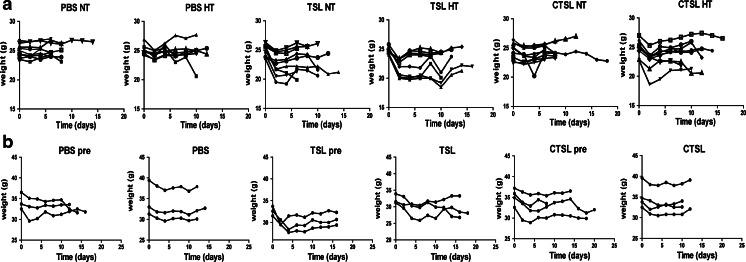
Body weight of the treated mice was followed every other day after treatment until death. (**a**) Body weight of mice with B16BL6 tumors. (**b**) Body weight of mice with LLC tumors.

Dox-TSL and Dox-CTSL effect on mice regarding their toxicity was tested by measuring body weight every other day after treatment. In B16BL6 tumor model, PBS and PBS plus HT treatment did not show any toxicity on mice. All the other treatments with or without HT demonstrated toxicity only in the first 2 days after treatment shown by drop in body weight. However, after 2 days, body weight of all mice from all treatment groups was recovered and remained stable until death (Fig. 4a). Similarly, in LLC tumor model, there was an initial body weight loss in all the tratment groups, which was recovered in 2 to 4 days after treatment and remained stable until death of mice (Fig. 4b).

### Intravital Microscopy on Liposome and Dox distribution in tumors

**Fig. 5:**
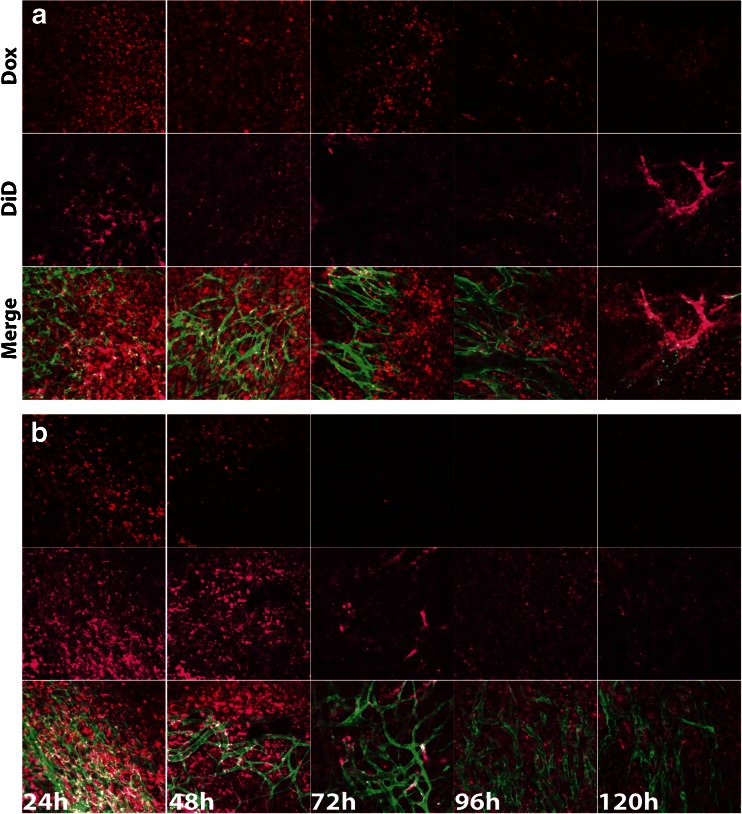
Doxorubicin and liposome retention in B16BL6 tumors implanted in window chamber bearing mice. Mice were injected with 5 mg/kg Dox-CTSL (**a**) or Dox-TSL (**b**) labelled with DiD. Liposomes were allowed to circulate for 5 h, after which HT was applied to the tumor for 1 h. Mice were observed up to 120 h in order to follow up the Dox and liposome clearance from the tumor.

In order to know which formulation of liposomes will be more effective in killing the tumor, we followed the retention of liposomes and Dox in tumor. Images of B16BL6 window chamber tumor bearing mice were taken up to 5 days after DiD labelled Dox-CTSL or Dox-TSL injection (Fig. 5). Images show that the two formulations extravasated from circulation 24 h after liposome injection and can be found vasculature or associated with it in the case of CTSL. Liposome and Dox clearance from the tumor progressed over time as can be concluded from decreased DiD and Dox fluorescent signal in the tumor. Remarkably, there was still an abundant amount of liposomes left in the tumor tissue even 120 h after injection of the two formulations. The Dox clearance from the tissue was faster for TSL (B) than for CTSL (A) which is suggestive for higher retention of CTSL in tumors.

### IHC of Tumor and Normal Tissues

**Fig. 6:**
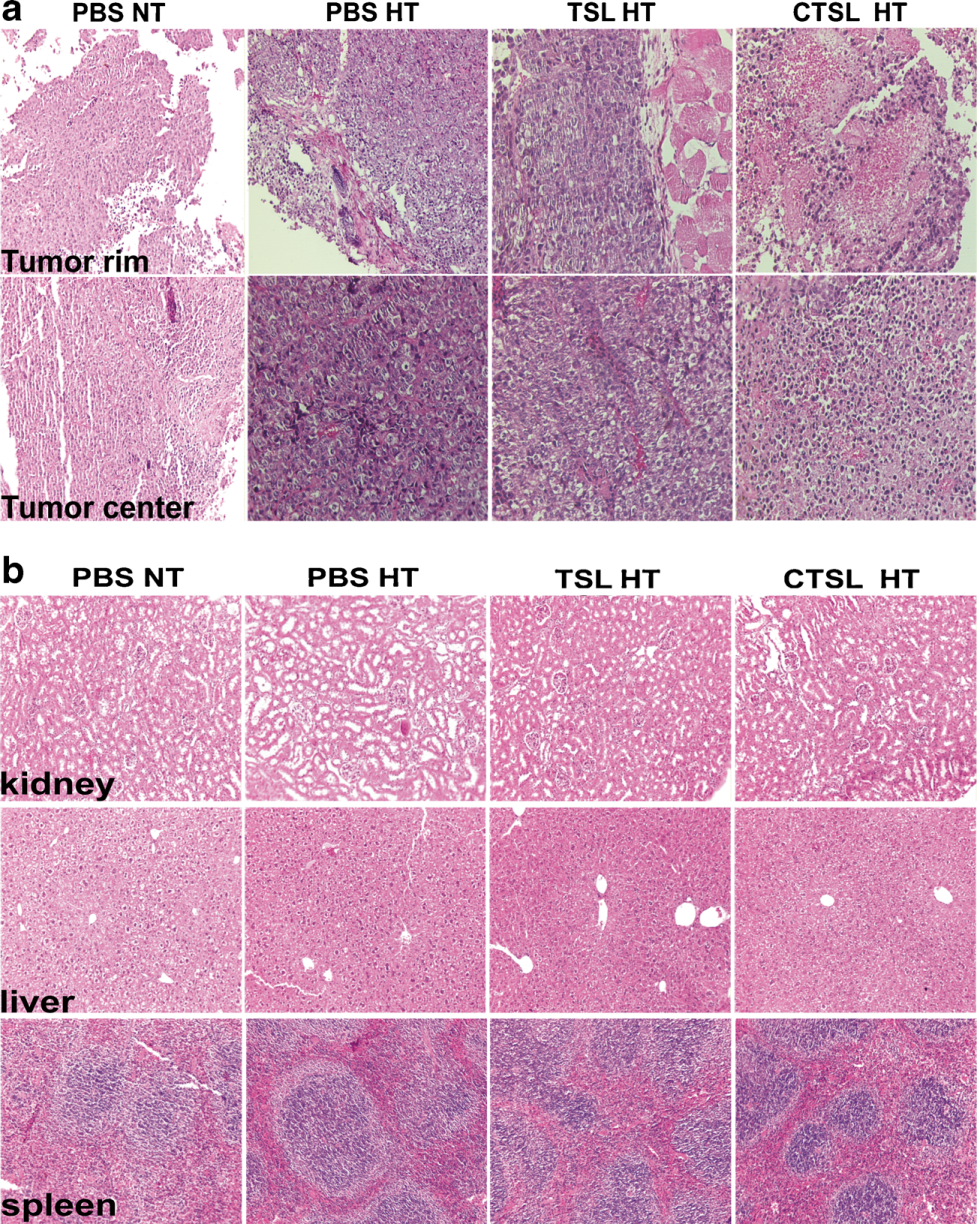
H&E staining of s.c. murine B16BL6 melanoma tissues (**a**) in tumor rim or tumor center and in normal tissues (kidney, liver and spleen) (**b**) treated with PBS, Dox-TSL or Dox-CTSL under HT.

Dox-CTSL plus mild HT for 1 h at 42°C caused interstitial haemorrhage in s.c. murine B16BL6 melanoma. Oedema was also seen in this treatment group. No obvious pathology was obserbed in tumors from mice from the other groups. None of the treatments showed any toxicity to the normal organs as concluded from the morphology of the spleen, kidneys and liver compared to the control PBS treatment (Fig. 6).

## Discussion

Thermosensitive liposomes loaded with Dox in combination with HT have emerged as a promising treatment approach for cancer patients (39–41). The aim of this approach is to increase drug levels in the tumor, thus increasing therapeutic efficacy. Several TSL formulations have been developed in the last two decades differing in their serum stability, thermosensitivity and ligand targeting (17,20,42–46). Liposome formulations having stability at 37°C and fast release kinetics at HT will offer best results in the clinic. For optimal application, stability of liposomes at physiological temperatures is very important in order to prevent premature drug release. Despite the many efforts made until now (42,47,48), the search for the best liposomal formulation is still ongoing. Reasons for this can be the limited tumor accumulation and specificity of liposomal nanoparticles, low drug bioavailability due to its stable entrapment and lack of control of drug release. Many novel approaches have been proposed in the past years to tackle these issues and thereby improve liposomal chemotherapy. We used two key approaches for improvement in this study being cell-specific targeting and temperature-controlled drug release. On one hand, CTSL are positively charged and will recognize negatively charged anionic sites on the membranes of tumor endothelial cells and tumor cells. In this way both tumor vasculature and tumor cells will be targeted. Once in contact, cationic lipids from CTSL will bind to anionic molecules on tumor or endothelial cells. This binding might evoke a receptor-mediated endocytosis, leading to CTSL internalization. Differently, CTSL are thermosensitive and when HT is applied drug release is forced, thus generating bioavailable drug either outside or within the targeted cell. The intracellularly released drug will then be transported to the nucleus where it can exert its therapeutic effect, while drug released outside the cell can either diffuse away or enter the cell.

Although previous studies focused on determination of Dox levels in tumors and blood and Al-Jamal *et al.* described the pharmacokinetics and biodistribution of different TSL (37), insight into biodistribution and pharmacokinetics of targeted TSL is lacking. Therefore, this study focuses on understanding these together with the therapeutic efficacy of our CTSL.

The pharmacokinetic behavior of Dox-TSL and Dox-CTSL was investigated with or without HT. In accordance with Al-Jamal *et al.* (37), local HT did not affect the blood kinetics of Dox from TSL and CTSL and it was cleared from circulation in a similar manner both under NT and HT conditions (Fig. 1a and b). After 1 h of liposome circulation under NT, ~50% of the encapsulated Dox was cleared, after which its concentration gradually decreased. This pharmacokinetic profile of CTSL proves that targeting does not cause faster liposome and subsequently drug clearance from circulation and it is in accordance with Dicheva *et al.* (43) showed by intravital microscopy that the concentration of fluorescently labelled CTSL and TSL in circulation was similar. Figure 5 confirms that targeting does not lead to faster liposomal clearance and shows that targeting contributes to a longer liposomal and drug retention in tumors. Dox from CTSL showed the only presence in circulation 4 h after injection, whereas Dox from TSL was completely cleared. At later time points, Dox levels were below detection in any of the formulations for both NT and HT.

Biodistribution studies showed that the highest uptake per gram of tissue of Dox-TSL and Dox-CTSL was in the spleen and the kidneys followed by the liver (Fig. 1b). This observation is also in accordance with Al-Jamal *et al.* (37) showing the highest uptake of their formulations in liver and spleen. The high spleen and liver uptake are due to the fact that these organs are part of the mononuclear phagocyte system (MPS), which is responsible for filtering out foreign particles from the blood circulation (49). There was no explanation why kidneys had an increased Dox uptake at both NT and HT conditions. Interestingly, HT increased Dox from CTSL in spleen, kidneys and liver. As expected and in accordance with Al-Jamal *et al.* (37), there was a little Dox uptake from the two formulations under NT and HT in heart and lungs and no uptake in brain and the leg muscle close to the heated tumor. The absence of Dox in the leg muscle shows that the heating was restricted only to the tumor. There was no difference in tumor uptake of Dox under NT from both formulations showing that targeting does not contribute to increased drug uptake at this condition. However, when initial HT for 1 h at 41°C was applied, there was an increased Dox uptake in the tumor from both formulations, which is likely due to increased extravasation of liposomes upon HT and therefore their higher accumulation at the tumor site. Additionally, Dox concentration from CTSL in the tumor was significantly higher compared to TSL, which is most likely due to the targeting nature of CTSL causing a higher accumulation of the carrier in the tumor and subsequently increased drug delivery.

In B16BL6 tumor model, HT itself had a tremendous effect in decreasing tumor growth (Fig. 3) but also increased survival as compared to only PBS treatment. HT as an additive treatment to liposomes had a great effect on CTSL in reducing tumor progression compared to TSL. In this case, the survival was increased from 8 to 12 days, whereas in the case of TSL the survival was not increased. There was not a significant difference in tumor growth inhibition between mice treated with Dox-TSL HT and Dox-CTSL HT, which shows that in this tumor model the targeting does not play a role in reducing the tumor volume compared with a non-targeted formulation.

As the efficacy study with B16BL6 did not show the benefit of using targeted thermosensitive liposomes in inhibiting tumor growth, LLC tumor model was included in a pilot study where two HT treatments were used—an initial mild HT at 41°C for 1 h to induce permeable tumor vasculature for liposome extravasation and; a second heat to trigger drug release (50). It was recently reported by Li *et al.* that a temperature of 41°C for 1 h can cause significant liposome extravasation in multiple murine and human tumor models (14). As seen in Fig. 3, the two HT treatments led to reduced tumor growth by Dox-CTSL compared to one HT treatment. The two HT treatments were most efficacious for Dox-CTSL showing increased survival from 10 to 18 days. Preheating phase had no effect on TSL and PBS when compared to one HT treatment.

Interestingly, histology demonstrated that only CTSL plus HT could cause hemorrhage and edema in the treated mice. This observation is in accordance with Dicheva *et al.* (43) demonstrating massive vessel destruction at 24 h after liposomal injection when CTSL are used in combination with HT. In the treated tumor models, HT showed the highest effect in tumor suppression as an additive to Dox-CTSL compared to Dox-TSL. This might be a result of its higher stability in serum leading to an increased levels of released drug upon HT. Another factor contributing to it might be that HT increases CTSL binding to endothelial cells (45) leading to its higher retention and effectiveness in tumor growth inhibition. However, more comprehensive studies about liposome pharmacokinetics are necessary. Interestingly, two HT treatments might have a better treatment result with targeted liposomes than one HT treatment. While intravascular release approach is considered to provide better results with non-targeted liposomes, the results presented here indicate a possible application for the so-called two step approach where HT is used to open up tumor vessels and to trigger release from targeted liposomes.

## Conclusion

Targeting of TSL did not lead to increased clearance of CTSL from circulation compared to TSL. Initial HT condition increased Dox uptake in tumors from CTSL compared to TSL. Efficacy study in B16BL6 tumor model demonstrated that HT had a significant effect on CTSL on tumor inhibition and prolonged survival. Efficacy study in LLC tumors showed that two HT treatments hold promises for successful therapeutic efficacy.

## Abbreviations

°C: Degree celsius
CTSL: Cationic thermosensitive liposomes
DMEM: Dulbecco modified eagle medium
Dox: Doxorubicin
DPPC: 1,2-dipalmitoyl-sn-glycero-3-phosphatidylcholine
DPTAP: 1,2-dipalmitoyl-3-trimethylammonium-propane
DSPC: 1,2-distearoyl-sn-glycero-3- phosphatidylcholine
DSPE-PEG2000: 1,2-distearoyl-sn-glycero-3-phosphoethanolamine-N-PEG2000
FCS: Fetal calf serum
Fig.: Figure
GFP: Green fluorescence protein
H: Hours
HEPES: 4-(2-hydroxyethyl)-1-piperazineethanesulfonic acid
HT: Hyperthermia
i.v.: Intravenous
Min: Minutes
Ml: Millilitre
NT: Normothermia
S.c.: Subcutaneous
SD: Standard deviation
SEM: Standard error
TSL: Thermosensitive liposomes
